# Genome-wide transcriptome analysis of the salt stress tolerance mechanism in Rosa chinensis

**DOI:** 10.1371/journal.pone.0200938

**Published:** 2018-07-26

**Authors:** Xiaoming Tian, Zhenyu Wang, Qing Zhang, Huacong Ci, Pengshan Wang, Lu Yu, Guixia Jia

**Affiliations:** 1 Beijing Forestry University, Beijing, China; 2 Tianjin TEDA Salina Eco-Landscape Research Center, Tianjin, China; Universidade de Lisboa Instituto Superior de Agronomia, PORTUGAL

## Abstract

Plants regulate responses to salt stress using biological pathways, such as signal perception and transduction, photosynthesis, and energy metabolism. Little is known about the genetics of salt tolerance in Rosa chinensis. Tineke and Hiogi are salt-tolerant and salt-sensitive varieties of *R*. *chinensis*, respectively, and are good choices for studying salt-tolerance genes. We studied leaf and root tissues from 1-year-old Hiogi and Tineke plants simultaneously grown under the same conditions. A 0.4%-mmol/L salt ion mixture was added to the basic growth medium. Illumina sequencing was used to identify differentially expressed transcripts. GO and KEGG pathway enrichment analyses were performed to identify differentially expressed genes. We identified many differentially expressed genes associated with salt tolerance. The abscisic acid-dependent signaling pathway was the main pathway that mediated the salt stress response in *R*. *chinensis*. Two pathways (plant hormone signal transduction and glutathione metabolism) were also active in salt stress responses in *R*. *chinensis*. The difference in salt tolerance in the cultivars was due to different gene sensitivity to salt in these two pathways. Roots also play a role in salt stress response. The effects of salt stress in the roots are eventually manifested in the leaves, causing changes in processes such as photosynthesis, which eventually result in leaf wilting. In Tineke, *Snrk2*, *ABF*, *HSP*, *GST*s, and *GSH1* showed high activity during salt stress, indicating that these genes are markers of salt tolerance.

## Introduction

Environmental stresses or abiotic factors, such as salinity, drought, heavy metals, and low temperature, often have a strong influence on plant survival and development. Plants have complex adaptive responses to abiotic stresses [1]. In their adaptation to salt stress, plants regulate many biological pathways, such as signal perception and transduction, photosynthesis, and energy metabolism [2]. Research on salt and drought has identified many stress-inducible regulators, including the upregulation of aquaporins, catalase, peroxidase and proline (PRO) accumulation [3]. In addition, abscisic acid (ABA)-dependent and -independent signaling pathways in plants are induced by stress [4]. Many transcription factors, such as ABA-binding factor (ABF) and drought-responsive elements are involved in the biological process of ABA [5]. Additionally, the accumulation of sodium and chloride in the cytoplasm results in cytotoxicity by reactive oxygen species (ROS) [6], which can lead to protein and lipid degradation and cell disruption [7]. Some salt-tolerant plant species have evolved mechanisms to cope with osmotic and ionic toxicity, including salt removal and extrusion of Na^+^ via an Na^+^/H^+^ antiporter in the vacuolar membrane [8]. Rosa chinensis (Chinese rose) is widely cultivated as an ornamental plant around the world. Although production of *R*. *chinensis* is increasing, environmental stress is an important factor limiting its growth. The successful cultivation of *R*. *chinensis* is influenced by salt stress [9], which is one of the most harmful factors affecting growth and productivity. Some studies have demonstrated the reduction of salt stress in *R*. *chinensis*, but more research is necessary. These studies have focused on petal development, flower bloom, and pests [10], but there is no information on the salt stress-related signaling pathways. Lack of molecular studies is an obstacle to understanding the molecular mechanisms underlying salt adaptation in *R*. *chinensis*. The development of next-generation sequencing technology has increased understanding of plant responses to drought and salt stress. Transcriptome sequencing techniques have been widely used in plant genomics research, including studies on the non-model plant tomato [11]. Therefore, we used RNA-seq technology to screen for salt-tolerance genes and to study the mechanisms of the salt stress response.

The Tineke rose is a salt-tolerant variety of *R*. *chinensis*, whereas the Hiogi rose is a salt-sensitive variety [12, 13]. The global transcriptome patterns in the roots and leaves of the two cultivars were compared to identify genes linked to salt tolerance. Sequence analysis revealed a large number of differentially expressed genes (DEGs) in *R*. *chinensis* under salt stress. Based on sequence similarities, we determined that genes related to salt responses and pathways are strongly represented within the DEGs.

## Materials and methods

### Plant material preparation and salt-stress treatment

Plant samples used were collected from the Tianjin Binhai New Area botanical garden with the approval of Tianjin Taida greening Group Co., Ltd. And no endangered or protected species involved in the experiment ground. One-year-old Hiogi and Tineke plants, planted at the same time and grown under the same natural environment conditions, were the study materials. Seedlings showing consistent growth were cultured in triangular cone flasks. Salt treatment was performed 10 d after normal growth conditions by adding a 1:1:3 ratio of 0.4% mmol/L NaCl : Na2SO4 : NaHCO3 to the base growth medium. Leaf and root changes were kept observing during salt stress. After 2 d the leaves began to wilt and the roots turned yellow and became soft. At 4 d, the leaves began to curl and the roots began to blacken and inactivate. At 7 d the leaves were yellow and the roots festering and at 9 d, the salt-sensitive Hiogi plants were dead, but the salt-tolerant Tineke plants remained alive. According to the phenotypic changes, the salt-tolerant and salt-sensitive properties can also be defined. Three young leaves and root tissues were taken for transcriptome sequencing at 2, 4, 7, and 9 d following salt treatment in Hiogi and Tineke, respectively (including 45 tissue samples, S1 Table). All fresh leaf and root tissues were frozen in liquid nitrogen and stored at −80°C for RNA extraction. For Hiogi, HL-0d, HL-2d, HL-4d, and HL-7d represent the control and salt treatment groups for each stage of the leaf tissue; HR-0d, HR-2d, HR-4d, and HR-7d represent the control and salt treatment groups for each stage of the root tissue. Thus, TL-0d and TL-2d, TL-4d, TL-7d and TL-9d represent the control and salt treatment groups for each stage in Tineke leaf tissue; TR-0d, TL-2d, TL-4d, TL-7d, and TL-9d represent the control and salt treatment groups for each stage of the root tissue.

### Determination of physiological indexes, photosynthetic gas exchange and tissue NaCl content

Water content, superoxide dismutase (SOD), PRO, and malondialdehyde (MDA) levels were measured in leaf and root samples. Chlorophyll a and b levels were also measured in leaves. Tissue water content was calculated based on fresh and dry weight. Total levels of SOD (LP-P0296, Lanpaibio, China), Pro (LP-P0296, Lanpaibio, China), and MDA (LP-P0296, Lanpaibio, China) were determined by ELISA (enzyme-linked immunosorbent assay) according to the protocol. Standard curves were established in accordance with the reagent instructions (S2 Table). Chlorophyll a and chlorophyll b levels were measured by spectrophotometric methods that were used in a previous study [14]. Five samples were taken as biological repeats in each group. Because the roots were ulcerated after 7 d of salt treatment, only the control roots, 2 d, and 4 d samples were used for measurement. Hiogi died after 7 d of salt treatment therefore, no physiological indexes were measured after 4 d. Tineke died after 9 d of salt treatment therefore we measured the physiological indexes of Tineke at 9 d. Photosynthetic rate (A), stomatal conductance (Gs), intercellular CO2 concentration (Ci) and transpiration rate (E) were measured using a LCpro-SD portable photosynthesis system (ADC Bioscientific Ltd., UK) according to previous studies [15, 16]. In addition, the NaCl content in leaf was also measured with an atomic absorption spectrophotometer. Briefly, the dried Rosa chinensis leaf samples were pulverized and grinded, and 0.5g was placed in muffle furnace at 650 C ashing for 8h. The ash was dissolved in 5% nitric acid and was fixed to 100ml with deionized water. The content of Na+ was measured using an atomic absorption spectrophotometer (AAS) (TAS-990, Purkinje, China). Standard curve was established with Na^+^ standard liquid (S3 Table).

### RNA extraction, library construction, and RNA sequencing

Total RNA extraction was performed using the TRIzol (Invitrogen, CA, USA) method, following the manufacturer instructions. RNA integrity was verified by 1% agarose gel electrophoresis and the concentration was measured using a Qubit Fluorometer (CA, USA). After RNA preparation, a total of at least 3 μg of RNA (treated with DNase) was used for the preparation of RNA sequencing libraries with a Lybay RNA Library Prep Kit (LB0014, Lybay, China). A total of 45 sequence libraries were constructed for each biological repeat sample. The Agilent Bioanalyzer 2100 system was used to assess library quality. Clustering of sequence libraries was performed on a cBot Cluster Generation System. After cluster generation, the enriched cDNA was sequenced using an Illumina Hiseq 4000 platform by Novogene Co., Ltd.

### RNA sequence assembly and DEG analysis

Clean, high-quality reads from eight samples were merged together and assembled using the Trinity package to construct unique consensus sequences as RNA-seq reference sequences. The annotation of the assembled transcriptome was performed with NR, NT, Pfam, COG, KEGG, and GO. The specific software parameter settings were obtained from previous research [17]. For sequence data in each sample, the EdgeR software package was used to statistically analyze the read counts, adjusted through one scaling normalized factor. Gene expression levels for each sample were determined using RSEM [18]. To obtain global gene expression patterns in different conditions, we performed pairwise comparisons using the DEseq for screening DEGs [19].

### Functional annotation and analysis of the transcripts

The annoated transcripts in NR database were further annotated with GO, and then classified into biological processes, molecular functions and cellular components using Blast2GO software [20, 21] according to previous studies [15, 22, 23]. After gene categories analysis, GO and KEGG enrichment were performed with GOseq software and KOBAS, respectively [24, 25]. GO terms (molecular function, cellular component, and biological process) and KEGG pathways with corrected p values (q value) of < 0.05 were considered significantly enriched. The KEGG Orthology database was used to statistically test the enrichment of DEGs in KEGG pathways.

### Validation of gene expression using reverse transcriptase real-time PCR (qPCR)

The qPCR experiment was performed using a CFX96 real-time PCR detection system (Bio-Rad, Hercules, USA) with three biological repetitions and three technical repetitions for each sample. The PCR reaction mixture (20 μl) included 75 ng template RNA, 0.4 μM primers, and 10 μl RNA-direct SYBR® Green Realtime PCR Master Mix, according to the manufacturer’s instructions. The total RNA used in qPCR was treated with DNase. *GAPDH* was selected as reference gene according to previous studies [20, 21]. And the expression of *GAPDH* was verified to be stable with the forward primer ‘CTCAGACTCCTCCTTGATAGC’ and reverse primer ‘TTCTGCCTGCTCTCAATGG’ (S4 Table). Relative gene expression levels were calculated using the 2^−ΔΔCT^ method, and regression analysis was performed between qPCR and RNA sequencing, including the salt tolerance candidate genes among Tineke and Hiogi.

## Results

### Effect of salt treatment on SOD, Pro, MDA, and other physiological indexes in *R*. *chinensis*

At the 2 d of salt treatment, the Tineke leaf showed slight curl and the root begins to turn yellow. But for Hiogi, the leaf was obviously curled and the root color turned yellow. At the 4 d of salt stress, the leaf in Tineke obviously curled with root color darkens. While the leaf in Hiogi start to turn yellow, and the root become black. At the 7 d, the Tineke leaf start to turn yellow, and the root died. For Hiogi, the leaf was withered with the root died. At the 9 d, only several leaves are alive in Tineke, and leaves on Hiogi were all died. The content of SOD, PRO, and MDA were measured base on standard curves. The result showed that SOD activity rapidly declined after 2^nd^ day of salt treatment in Hiogi, but in Tineke, until 4 days salt treatment later there showed a precipitous decline in SOD (Table 1). The PRO and MDA concentrations of HL dropped firstly then raised after the 7th day. For TL, the PRO and MDA showed a significant decline (*p*<0.05) in 2^nd^ day and then increased after 4^th^ day and maintained a relatively stable content. For root tissue, both of PRO and MDA showed a continuous decreasing trend with the increase of salt treatment time. After salt treatment, water content significantly decreased (*p<0*.*05*) in all tissues, and in the later stage of salt treatment, the trend of decline tended to be gentle. The water content in Tineke root was higher than that in Hiogi in the 4d salt treatment. Chlorophyll a and b levels in Hiogi significantly decreased (*p* <0.05) after 2 d of salt stress, but the levels of Tineke significantly decreased (*p*<0.05) only after 4 d.

**Table 1:** Concentrations of physiological indexes.

	SOD Content (pg/g)	PRO Content (ng/g)	MDA Content (ng/g)	Water content (%)	Chlorophyll a (mg/g)	Chlorophyll b (mg/g)
HL-0d	952.63	57.29	10.47	78.91	1695.99	629.94
HL-2d	72.56	32.03	3.19	73.02	1428.78	608.63
HL-4d	58.78	28.67	2.73	65.54	983.94	597.49
HL-7d	64.39	34.10	4.18	53.33	913.88	585.70
HR-0d	927.84	51.98	7.27	90.21	—	—
HR-2d	91.03	30.15	3.11	82.66	—	—
HR-4d	80.29	25.32	2.41	78.03	—	—
TL-0d	1032.35	63.56	9.08	82.10	1648.40	827.74
TL-2d	805.71	30.85	2.92	69.33	1632.52	729.67
TL-4d	59.32	31.02	5.67	60.17	1007.03	489.15
TL-7d	57.19	33.19	5.40	57.33	918.44	526.29
TL-9d	56.03	33.75	6.05	57.67	630.90	288.78
TR-0d	829.42	49.07	9.48	90.14	—	—
TR-2d	661.95	33.17	4.26	85.15	—	—
TR-4d	88.29	30.99	3.99	84.67	—	—

### Effect of salt treatment on photosynthetic gas exchange and NaCl content in leaf

**Fig 1:**
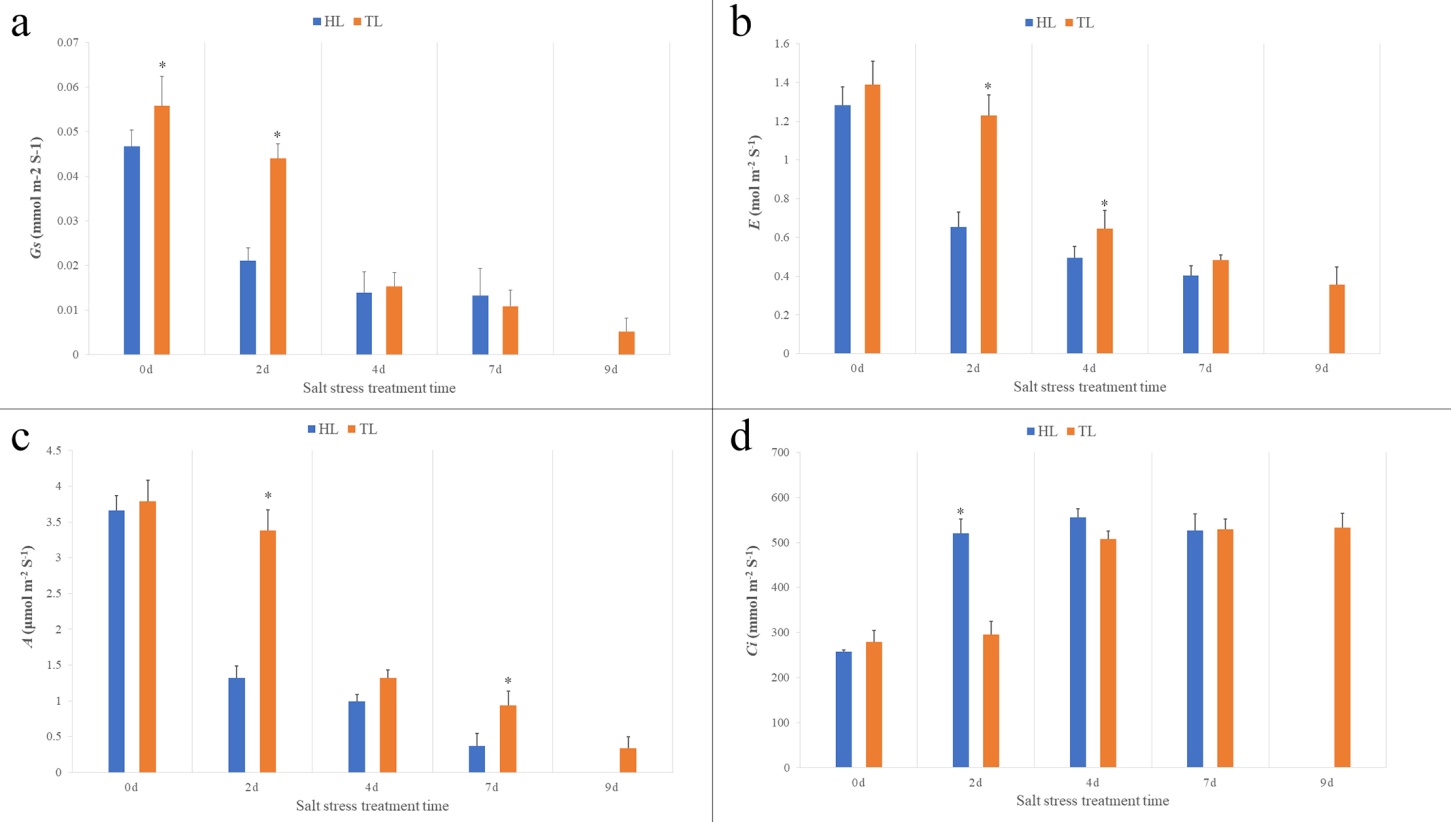
Photosynthetic gas exchange in different salt stress treatment time. a: Stomatal conductance (*Gs*), b: Transpiration rate (*E*), c: Photosynthetic rate (*A*), d: Internal CO2 concentration (*Ci*). The values are means +SD (Standard Deviation) (n = 5). The asterisk indicates a significant difference (*p*<0.05).

**Fig 2:**
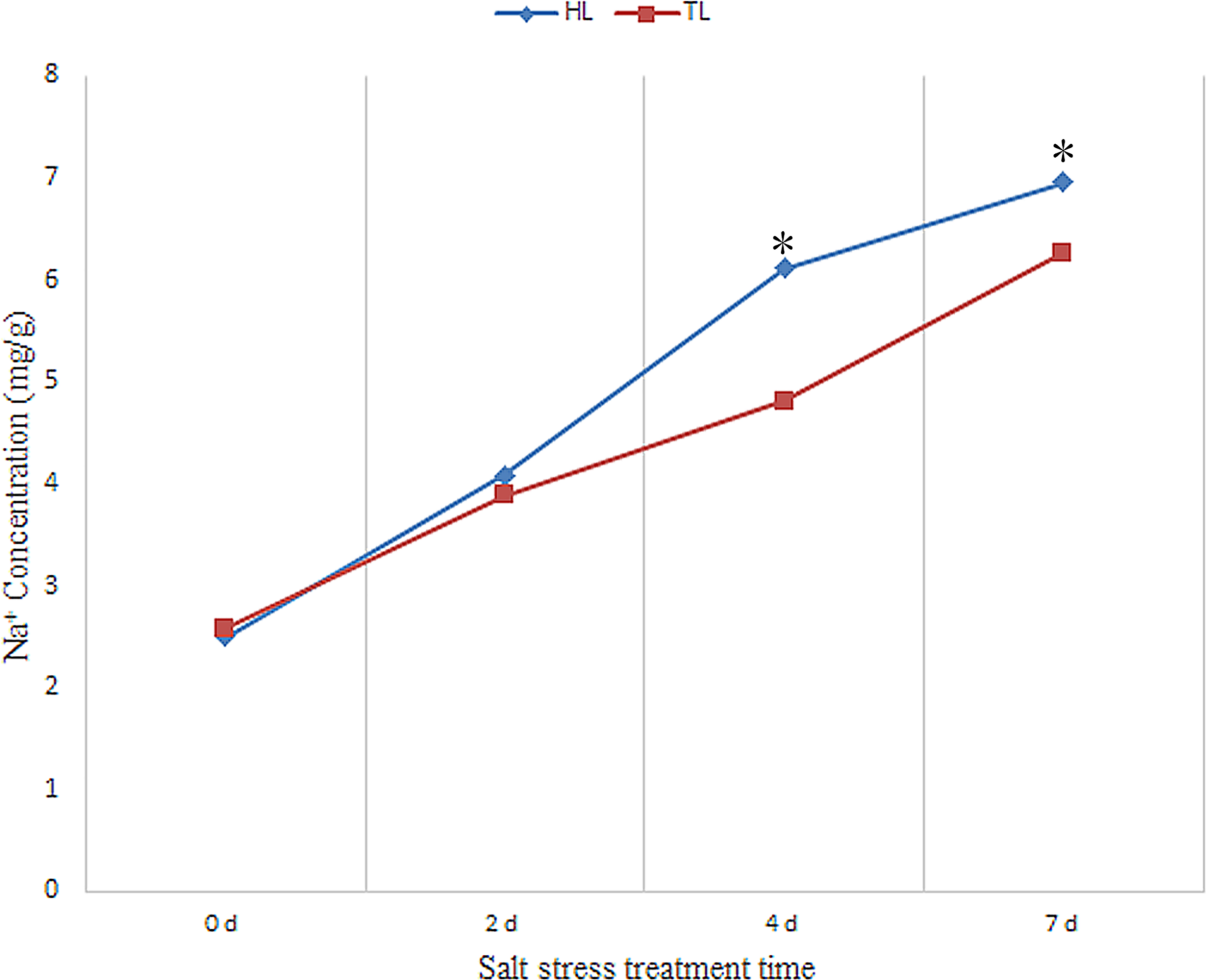
Na^+^ content in leaf tissue. The values are means (n = 5). The asterisk indicates a significant difference (*p*<0.05).

Photosynthetic gas exchange is an indication of plant health status, by which the salt stress damage to plants can be indirectly reflected. The photosynthesis rate (*A*), stomatal conductance (*gs*) and transpiration rate (*E*) reduced with the increasing salt treatment time (Fig 1A to 1C). The internal CO2 concentration (*ci*) increased with the increasing salt treatment time (Fig 1D). For Hiogi, the photosynthesis rate, stomatal conductance and transpiration rate sharply reduced in the 2^nd^ day of salt treatment. While for Tineke, the sharply reduction occurred in the 4^th^ day of salt stress. For the internal CO2 concentration, there were little changes after 2 days and 4 days salt treatment in Hiogi and Tineke, respectively. In addition, the NaCl content in leaf showed that the change trend of NaCl concentration, with the increase of salt stress time, were different between the two rose varieties (Fig 2). For Hiogi, the concentration of NaCl raised sharply after 2d. For Tineke, with the increase of salt treatment time, the concentration of NaCl gradually increased. At 4d and 7d, the NaCl content in Tineke was significantly lower than that in Hiogi.

### RNA sequencing, assembly, and annotation

**Fig 3:**
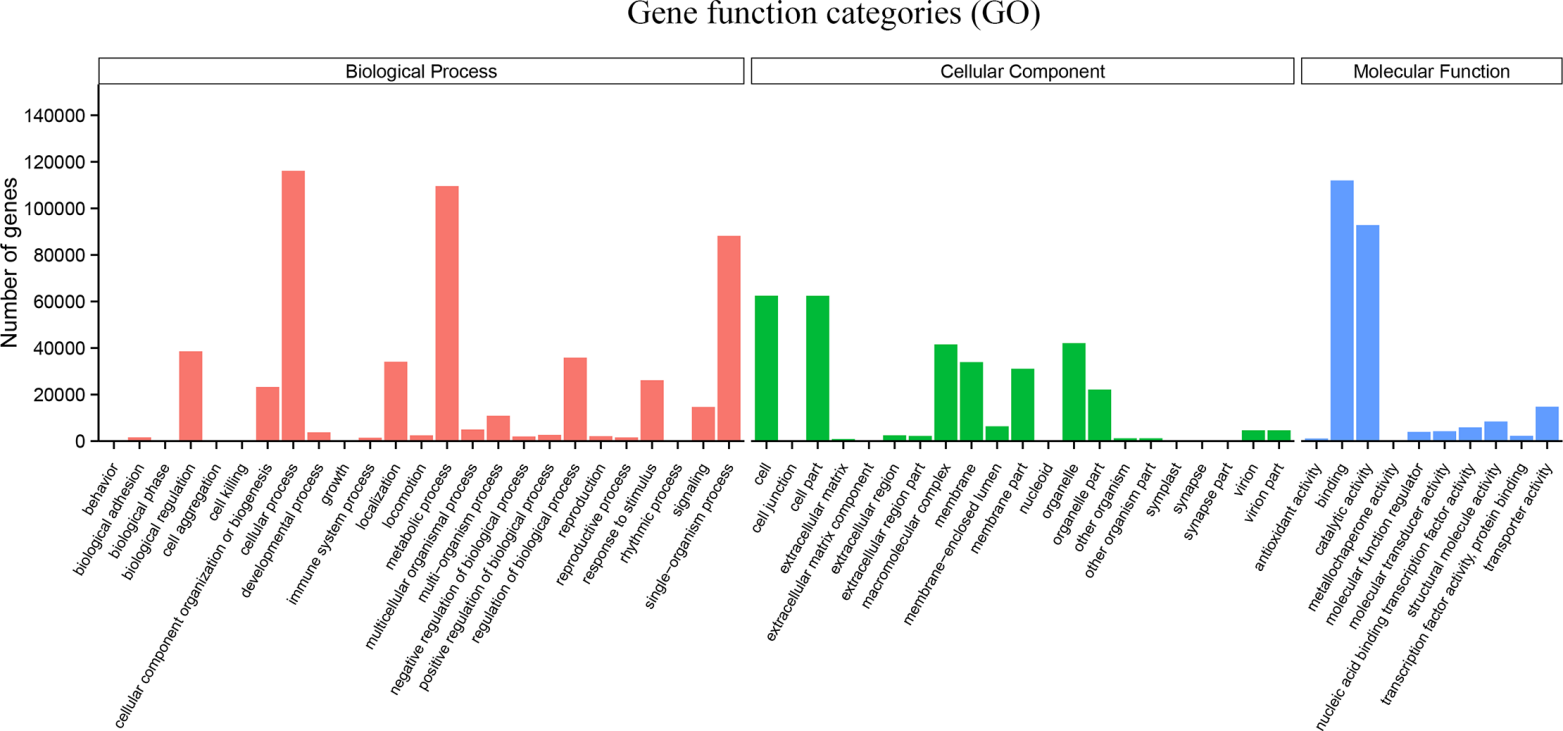
Gene function categories in GO.

A total of 168.97 GB of raw data from the 45 libraries was generated with an average sequencing error rate of 0.02% and an average GC content of 45.98% (S5 Table). The data ratio of sequencing quality to Q20 and Q30 was 96.90% and 92.19%, respectively. All sequence data were uploaded to the SRA database with the SRA accession number SRP128235. As *R*. *chinensis* did not have an appropriate reference genome sequence, the Trinity method was used to assemble, *de novo*, all of the clean reads. In total, 1,126 million high-quality reads were assembled into 465,404 unigenes with an N50 length of 1,302 bp and an N90 length of 443 bp (Table 2). To obtain a general idea of the total sequenced mRNA, the transcripts were searched against the Nr, Nt, Pfam, KOG, Swiss-Prot, KEGG, and GO databases. Altogether, 319,798 unigenes were successfully matched with at least one database, with 239,038 (51.36%), 214,867 (46.16%), 102,631 (22.05%), 205,637 (44.18), 205,752 (44.2), 207,678 (44.62%), and 89,076 (19.13%) annotated transcripts (17.15%) showing a significant hit against the Nr, Nt, KEGG, Swiss-Prot, Pfam, GO, and KOG database, respectively. Functional annotation showed that biological processes, molecular functions, and cellular component categories occupied 47.98%, 29.42%, and 22.60% of the total sequence, respectively, including the annotated terms response to stress, response to abiotic stimulus, response to stimulus, and other terms. Based on a comparison with the KEGG database, 111,229 (34.78%) of the 319.798 annotated unigenes had significant matches and were thus assigned to 130 KEGG pathways, including “carbon metabolism” (5,455 unigenes), “ribosome” (6,549 unigenes), “purine metabolism” (2,460 unigenes), and “plant hormone signal transduction” (1,342 unigenes), which were all related to probable resistance responses. The GO classification results showed that 1,086,389 transcripts were divided into 3 categories: 521,204 (47.98%) in biological process, 319,655 (29.42%) in cellular components and 245,530 (22.60%) in molecular function (Fig 3). The GO classification analysis showed that these transcripts were mainly involved in the biological processes including signaling, negative regulation of biological process, response to stimulus, and cellular process; For cellular components, cell, cell part, and organelle were the top three GO terms; For molecular function categories, binding, catalytic activity, and transporter activity were mainly involved.

**Table 2:** Summary of transcriptome assembly.

Category	Number				Total number	Mean length (bp)	N50 (bp)	N90 (bp)
	200–500 bp	500bp–1kb	1–2 kb	>2 kb				
**transcripts**	845713	170289	91293	46459	1153754	539	771	234
**unigenes**	167193	160553	91199	46459	465404	941	1302	443

### DEG identification and GO and KEGG functional analysis in leaf tissue

**Fig 4:**
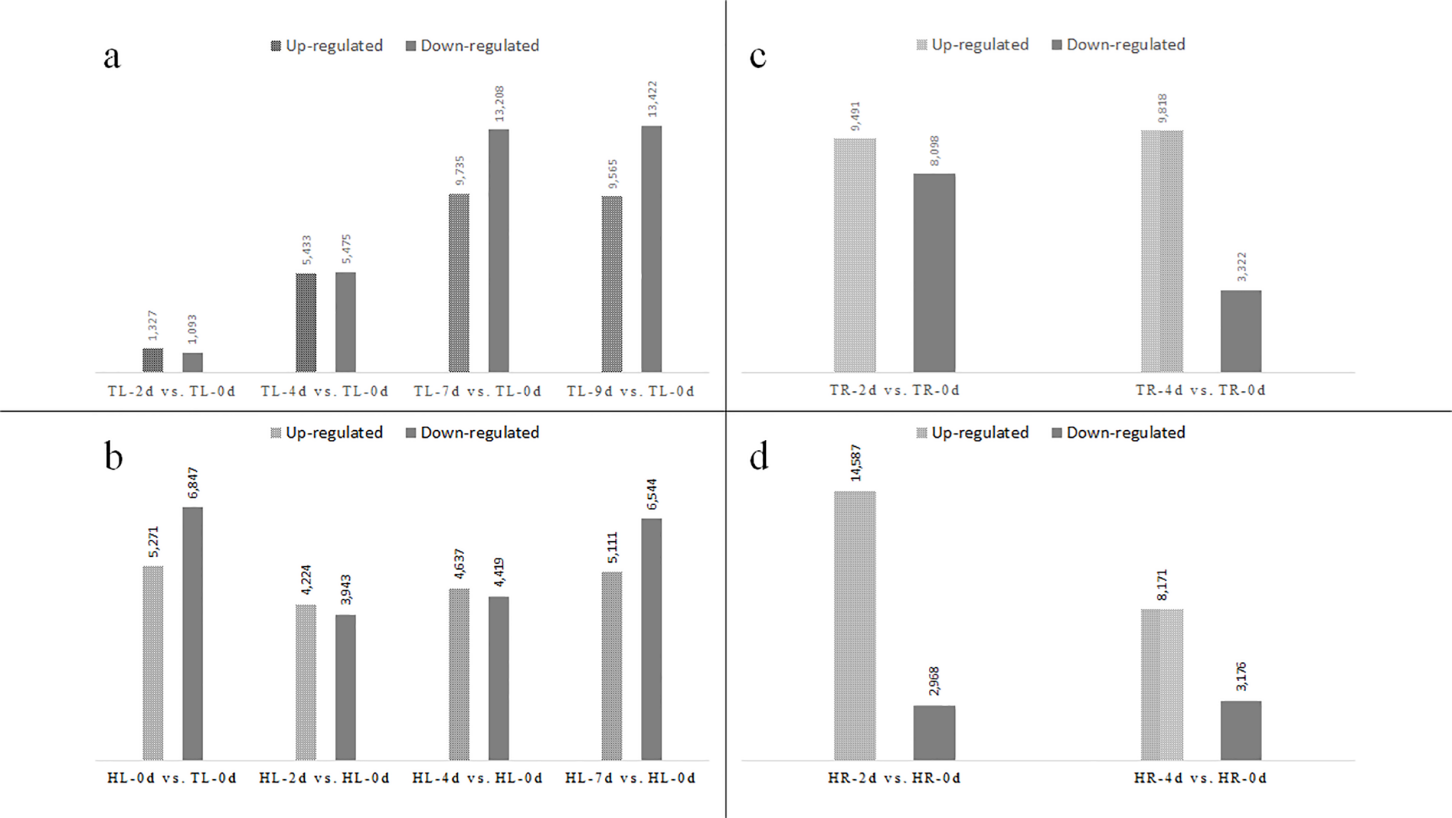
Number of DEGs in Rosa chinensis. Number of differential genes between different comparisons. a. Number of DEGs in *Tineke* leaf; b. Number of DEGs in *Hiogi* leaf; a. Number of DEGs in Tineke root; b. Number of DEGs in Hiogi root.

**Fig 5:**
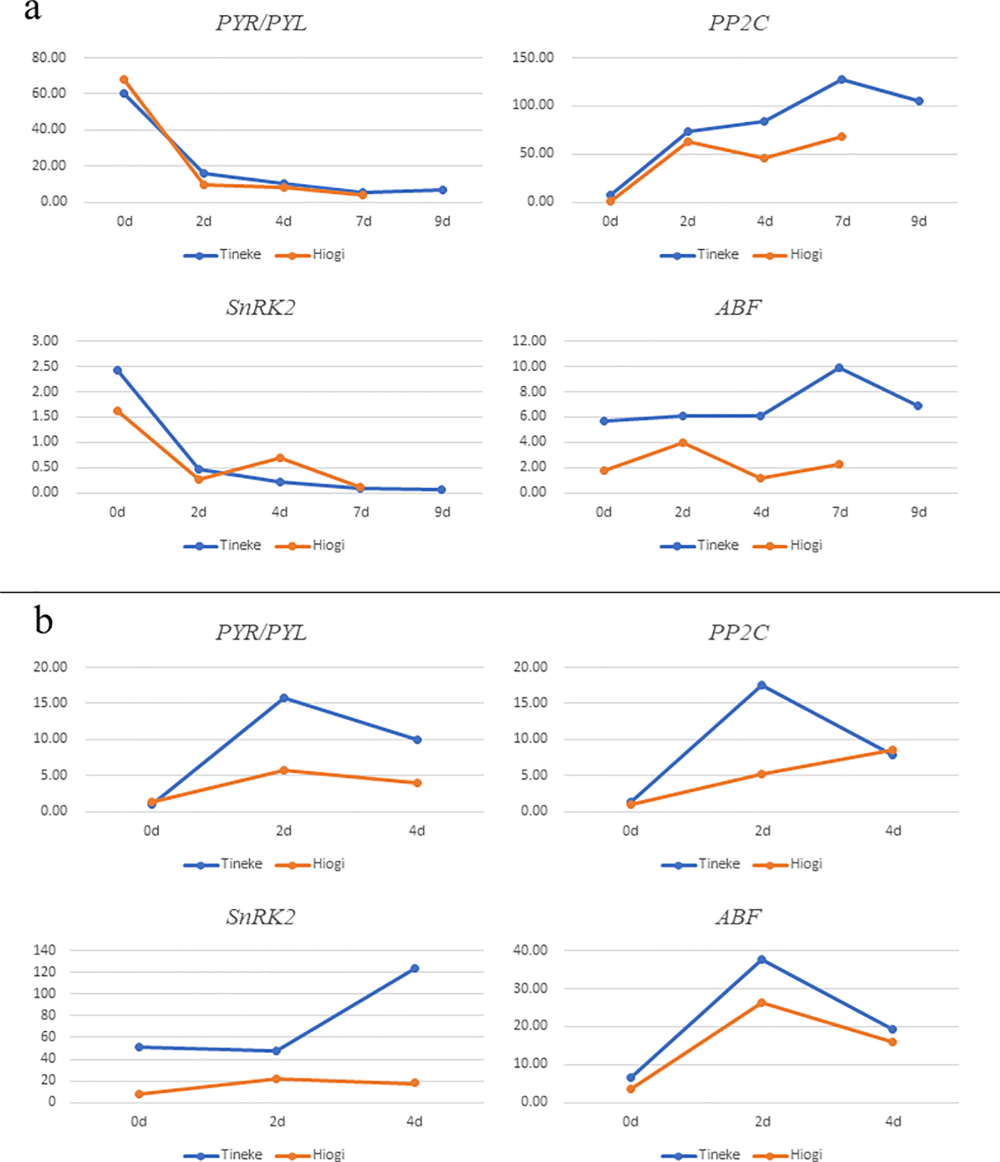
Expression of *PYR/PYL*, *Snrk2*, *PP2C*, and *ABF*. a. *PYR/PYL*, *Snrk2*, *PP2C*, and *ABF* expression in leaf tissue. b. *PYR/PYL*, *Snrk2*, *PP2C*, and *ABF* expression in root tissue.

Comparison between Tineke and Hiogi was performed for the untreated and salt-treated groups. Among DEGs in HL-0d vs. TL-0d, HL-2d vs. TL-2d, HL-4d vs. TL-4d, and HL-7d vs. TL-7d, 1,734 DEGs overlapped (S1 Fig). There were 5,271 up regulated DEGs and 6,847 down regulated DEGs in HL-0d vs. TL-0d including *PYR/PYL*, *PP2C*, *HEXA*, *crtB*. In addition, ADP binding, protein binding, estrogen metabolic process, and steroid dehydrogenase activity were all enriched base on the down-regulated DEGs but no GO terms enriched base on up-regulated DEGs. This indicates that Tineke had higher activity in ADP binding, protein binding and other molecular function. The KEGG enrichment showed that Porphyrin and chlorophyll metabolism, Carotenoid biosynthesis, Plant hormone signal transduction, and RNA degradation were significantly enriched (p<0.05) base on the up regulated DEGs in HL-0d vs. TL-0d, which reveal that the expression of DEGs in these pathways of Hiogi were higher than that of Tineke. In the process of salt stress treatment, Hiogi was more affected than Tineke because the expression of unigenes in Hiogi, but not in Tineke, greatly changed in the first 2 d, and this continued until 7 d (Fig 4A). In contrast, the number of DEGs in Tineke slightly increased in the first 2 d and then increased with the continuation of salt treatment until 9 d (Fig 4B). This result indicates that the Tineke response to salt stress involved maintenance of a relatively stable internal state. There were more DEGs expressed in roots than in leaves (Fig 4C and 4D). In the comparison of HR-0d vs. TR-0d and HR-2d vs. TR-2d, 18,121 and 21,612 DEGs were found, respectively. DEGs in the comparison of HR-0d *vs*. TR-0d including *MYC*, *ETR*, *LOX*, and *WRKY*. The KEGG enrichment analysis showed that plant hormone signal transduction, ADP binding, protein binding, RNA degradation, and thiamine metabolism were the prominent pathways. Through the data, the expression pattern showed large difference in root tissue. Plant hormone signal transduction and ADP binding were the most important difference between the two Rosa chinensis varieties. For HR-4d vs. TR-4d, only 5,154 DEGs were found (S2 Fig). The DEG results showed that the numbers of upregulated and downregulated genes were similar in Tineke after 2 d of salt treatment and the greatest difference in the number of upregulated and downregulated genes occurred at 4 d. For Hiogi, the number of upregulated genes was much higher than the number of downregulated genes at 2 d of salt treatment (Fig 5). Among all the DEGs mentioned above, 2,132 DEGs overlapped including heat shock protein (HSP), CYP450, and ERFs, which were highly expressed in Tineke. The glutathione synthetase-related gene glutamate–cysteine ligase (*GSH1*) was highly expressed in Tineke, which enhanced the activity of the GSH metabolism pathway. Most DEGs were associated with secondary metabolites in the roots and some were annotated as transcription factors, protein modification and degradation factors, and phytohormone response factors.

GO analysis showed that 25 and 7 GO terms were significantly enriched (*p*<0.05) in HL-0d vs. TL-0d and HL-2d vs. TL-2d, respectively, and no terms were enriched at 4 d and 7 d. The anaphase-promoting complex and the nuclear ubiquitin ligase complex were included. There were 502, 131, 143, and 113 enriched GOs in HL-2d, HL- 4d, and HL-7d when compared with HL-0d. The top 20 overlapped GOs included those related to catalytic activity, drug transporter activity, and oxidoreductase activity (S6 Table). In Tineke, 113, 210, 220, and 214 GOs were enriched in TL-2d, TL-t 4d, TL-7d, and TL-9d compared to the control. The most affected GO terms in Hiogi were related to catalytic activity, metabolic processes, oxidoreductase activity, and single-organism processes. The KEGG pathway enrichment result showed that 10, 12, 6, and 7 pathways were enriched in HL-0d, HL-2d, HL-4d, and HL-7d, respectively, compared with the corresponding time points in Tineke. Two pathways overlapped, including plant hormone signal transduction and carotenoid biosynthesis (S7 Table). Photosynthesis-related pathways were also significantly enriched (*p≤0*.*05*) in DEGs in Hiogi and Tineke, and the related genes were highly expressed in Hiogi. Moreover, 28, 22, and 29 significantly enriched (*p<0*.*05*) KEGG pathways were found in HL-2d, HL-4d, and HL-7d, respectively, when compared with TL-0d, including those related to plant hormone signal transduction and starch and sucrose metabolism.

### Plant hormone signal transduction changes after salt treatment in *R*. *chinensis*

**Fig 6:**
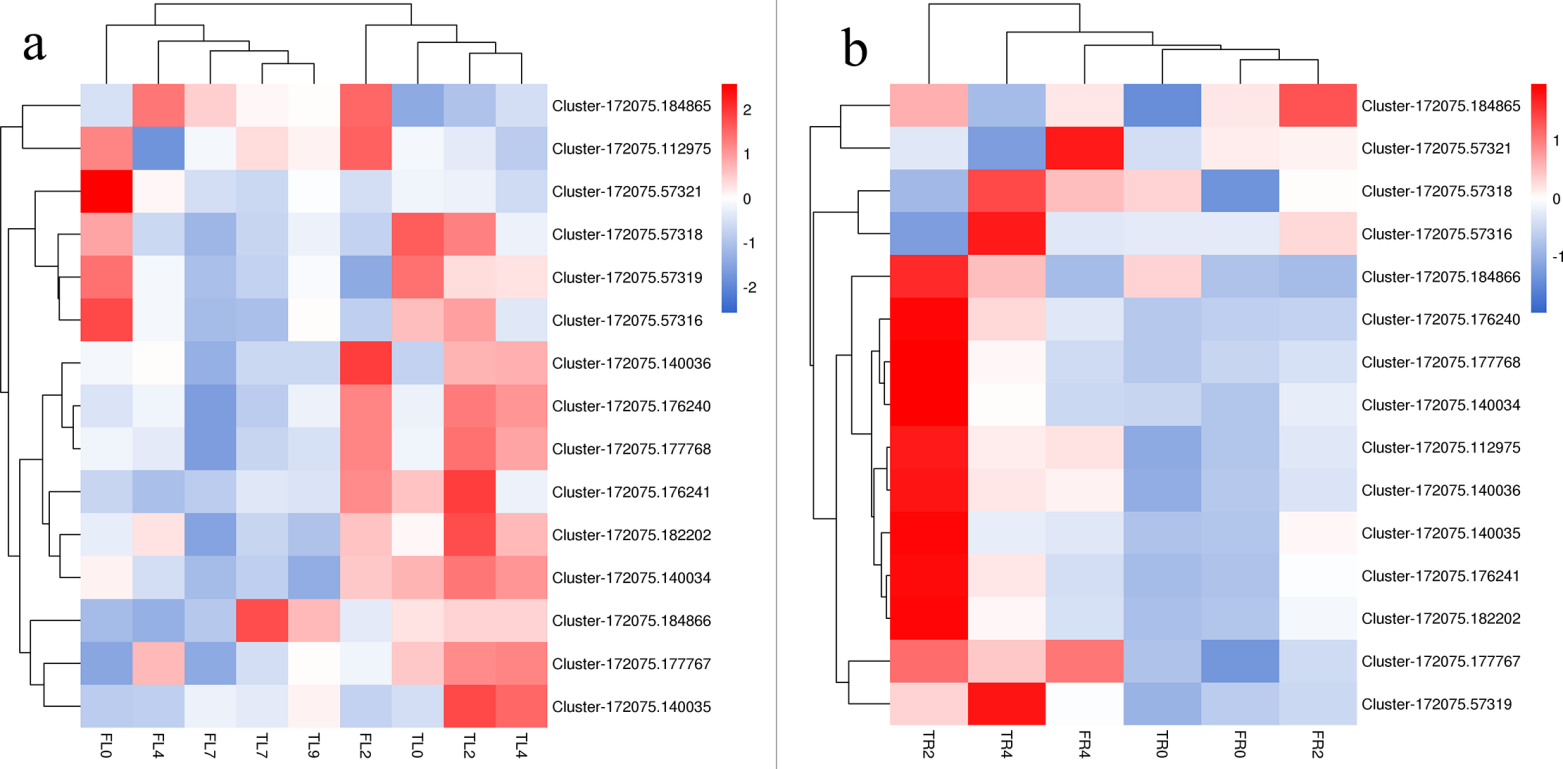
DEGs expression heatmap of transcripts of *ABFs*. a. DEGs expression heatmap of transcripts of *ABFs* in leaf tissue. b. DEGs expression heatmap of transcripts of *ABFs* in root tissue.

KEGG enrichment results showed that the plant hormone signal transduction pathway was significantly enriched (*p<0*.*05*) in the leaves and roots of the two *R*. *chinensis* varieties. The most obvious changes were to the ABA signal transduction process, and there were great differences in *PYR/PYL*, *PP2C*, *Snrk2*, and *ABF* between the different varieties. Fig 5 shows the expression pattern of these four genes in the leaves and roots of Hiogi and Tineke. *PYR/PYL* and *Snrk2* showed a similar expression trend in all leaf tissues. *PP2C* expression level was higher in Tineke after 4 d of salinity stress, and the key transcription factor *ABF* expression level was also higher in the leaf tissue of Tineke compared to the leaf of Hiogi. For root tissue, the expression of *PYR/PYL*, *Snrk2*, *PP2C*, and *ABF* in the early salt treatment stage was significantly higher (*p<0*.*05*) in Tineke, and 4 d later, there was no significant difference in *PP2C* expression between Hiogi and Tineke. Fifteen transcripts of *ABF* were found and heatmaps were constructed to illustrate the expression pattern of *ABF* (Fig 6). Most of the *ABF* transcripts that were upregulated in Tineke leaves were downregulated (or not significantly different) in Hiogi. In roots, the expression level of these 15 transcripts in Tineke were significantly higher (*p<0*.*05*) than that in Hiogi and, at 2 d, the mRNA expression level was the highest in Tineke.

### Glutathione metabolic changes after salt treatment in *R*. *chinensis*

**Fig 7:**
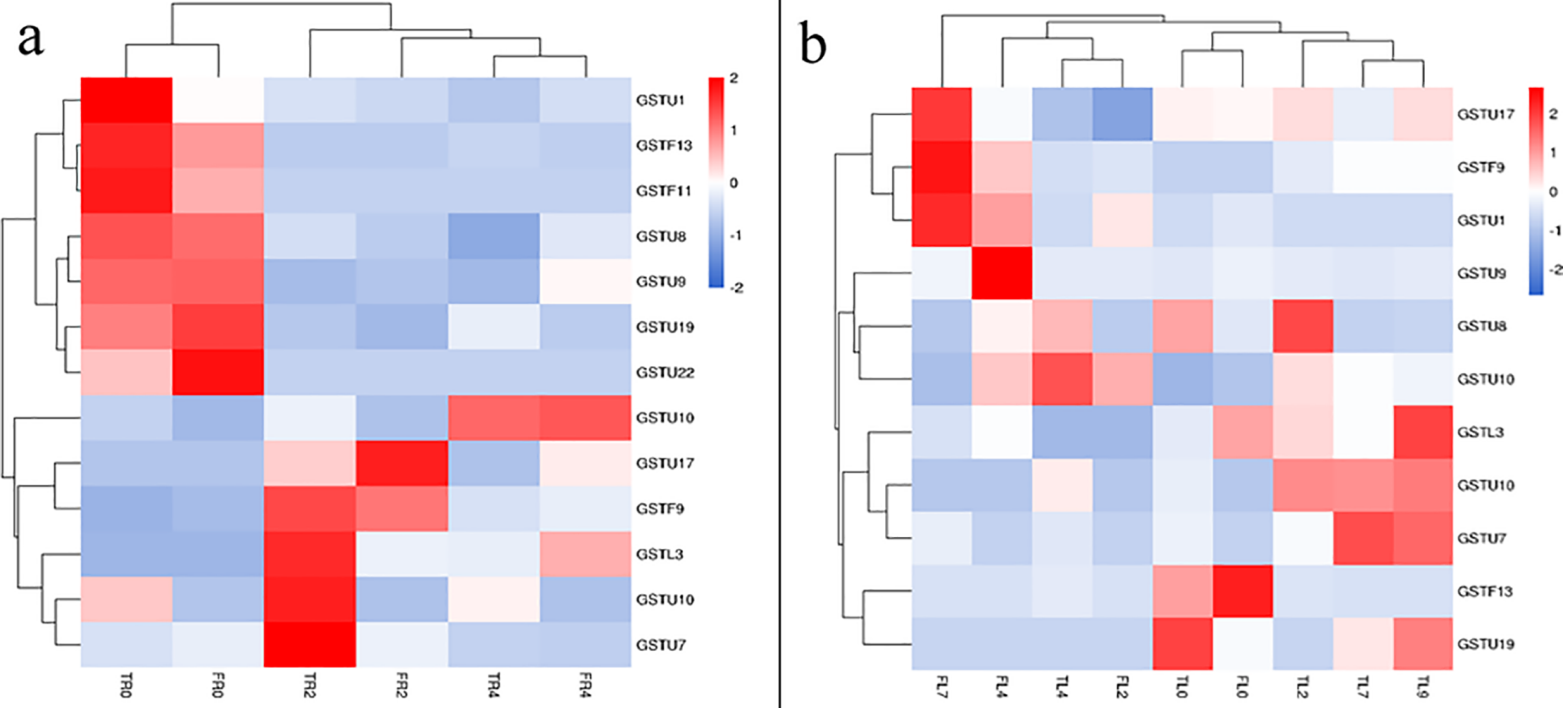
DEGs expression heatmap of members of *GSTs* falimy. a. DEGs expression heatmap of members of *GSTs* falimy in leaf tissue. b. DEGs expression heatmap of members of *GSTs* falimy in root tissue.

The glutathione metabolic pathway was significantly enriched (p<0.05) in both the leaves and roots of Tineke, but there was no significant enrichment in Hiogi (Table 3). In leaf tissue, the number of DEGs increased with increasing salt treatment time. The functional annotation of DEGs showed that most APX family members (*APX6*, *APX3*, *APX2*, *APXT*) were specifically upregulated in Tineke leaves and roots. In addition, 10 GST family members (*GSTU1*, *GSTU8*, *GSTF11*, *GSTF13*, *GSTU9*, *GSTU19*, *GSTU22*, *GSTU17*, *GSTU10*, *GSTF9*, *GSTL3*, *GSTU7*, and *GSTU10*) were differentially expressed after salt treatment. Some of these members were upregulated after salt treatment and some were downregulated (Fig 7). *GSH1* plays an important role in glutathione synthesis. *GSH1* expression was upregulated after salt treatment. For Hiogi, relatively few genes related to the glutathione metabolic pathway changed significantly (*p*<0.05) in expression. There were few differentially expressed *GSTs* in Hiogi after salt treatment (Table 3).

**Table 3:** DEGs in glutathione metabolic pathway.

DEG number	Background number	P-value	Corrected P-value	Comparation
33	1191	0.554203	1.00	HL2 vs HL0
17	1191	0.117473	0.36	HL4 vs HL0
54	1191	0.111765	0.34	HL7 vs HL0
15	1191	0.047709	0.16	TL2 vs TL0
65	1191	0.000286	0.00	TL4 vs TL0
116	1191	0.001337	0.01	TL7 vs TL0
107	1191	0.003009	0.01	TL9 vs TL0
71	1191	0.994919	1.00	HR2 vs HR0
63	1191	0.005869	0.06	HR4 vs HR0
151	1191	2.61E-06	0.00	TR2 vs TR0
68	1191	0.007885	0.01	TR4 vs TR0

### Verification of transcript expression levels using qPCR

**Fig 8:**
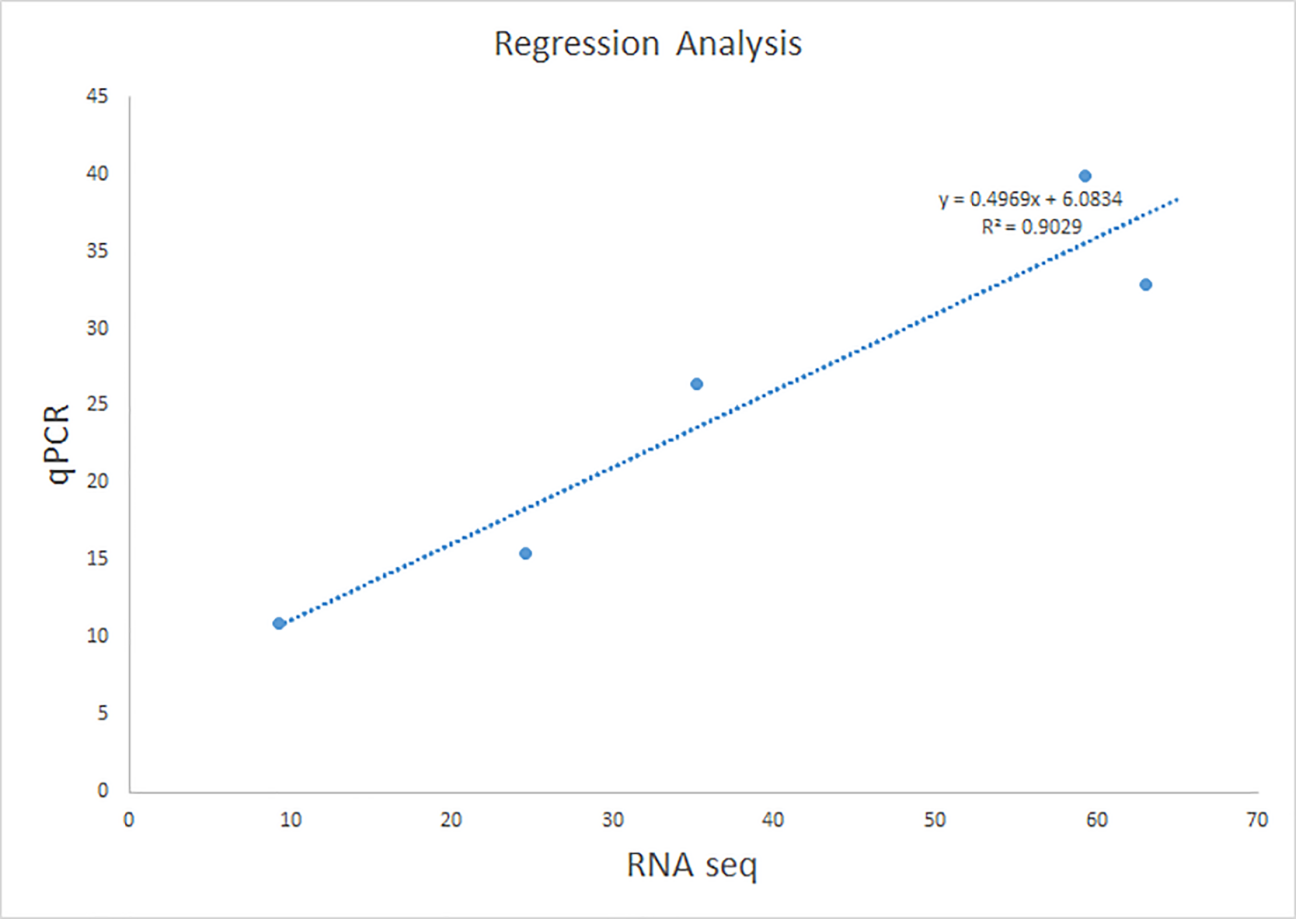
Correlation between qPCR and RNA sequencing for the 5 selected genes. Each point represents a value of fold change of expression level.

Five transcripts of *PYR/PYL*, *PP2C*, *Snrk2*, *GSH1*, and *ABF* were quantified by qPCR. All primers designed for qPCR are shown in Table 4. The qPCR results showed that gene expression patterns were consistent with transcriptome sequencing. *PYR/PYL* and *Snrk2* showed similar expression in Tineke and Hiogi. With increased salt treatment time, the fold change of expression decreased. However, *PP2C* showed higher expression in Tineke than in Hiogi after 4 d of salt treatment. In addition, the fold changes of *ABF* expression in Tineke was also significantly higher (*p<0*.*05*) than that in Hiogi. This result shows that *GSH1* was highly expressed in Tineke, whereas in Hiogi there were no obvious changes in *GSH1* expression after salt treatment. The salt stress-induced changes in gene expression for the majority (~70%) of the tested genes were consistent between both approaches (RNA-seq and qPCR). The correlation coefficient (r^2^) between qPCR- and FPKM-derived expressions was 0.9029 (Fig 8), indicating high reliability of the RNA-seq data.

**Table 4:** Primers.

Gene	Primer sequences (5ʹ-3ʹ)	Annealing temperature (°C)	Product length (bp)
*PYR/PYL*	F: TTGGCAAGAAGTCAACAAACAG	57.5	146
	R: CACAGAAAAGATGGCTGGTAAC	57.5	
*PP2C*	F: TAGTTGTCACCTATGGCTCTTG	57.5	137
	R: CCTCTTTCCTCCGATCACAAG	57.5	
*SnRK2*	F: TTGGGAGTTGAAATGGATCG	57.5	141
	R: GGTCTGCTTGTCCGTCATC	57.5	
*ABF*	F: AGGTTATCACAGAAGCCATCC	57.5	138
	R: CCAGTTTATTGCTGCCTCTTG	57.5	
*Gsh1*	F: ATTCCAGGCTCTTCACTTTCC	57.5	141
	R: GTTTGGGTTCTTTGTTCAGG	57.5	

## Discussion

Plants typically accumulate small molecules, organic compounds, PRO, and protein protective agents in their cells to maintain osmotic homeostasis (PRO and glycine betaine) when subjected to salt stress [26, 27]. In this study, the contents of PRO and MDA in Tineke leaf rose earlier than Hiogi, but the accumulation of PRO and MDA did not reach the level before salt stress treatment. This was not in line with the laws of other plants [28–30]. This might be that the response to salt stress in Rosa chinensis did not depend on PRO and MDA. The rise of PRO and MDA content indicated that they also play a role in salt tolerance, but obviously not the most critical role. Also, the PRO and MDA result reflected the stronger tolerance of Tineke to salt stress. In addition, the NaCl result also proved that the NaCl contents regulation ability in Tineke was also better than that in Hiogi. Under salt stress, plant roots firstly receipt salt stress signal [31]. Root was affected by high permeability and caused the loss of water content [32]. The loss of water in roots further caused serious damage to the plants. The changes of water content in this study indicated that Tineke can control the loss of water better than Hiogi, which was is consistent with the salt tolerance characteristics of Tineke. Recent studies have demonstrated gene expression cascades in plants under abiotic stress. This is a defining feature of the ABA-dependent and ABA-independent signaling pathways and their association with stress responses [33]. After 2 d of salt treatment, plant hormone signal transduction was induced by the salt and played an exclusive role in Tineke and Hiogi responses to salt stress. *PYR/PYL* is a receptor of the ABA-signaling complex, and overexpression of *PYR/PYL* could suppress *PP2C*s, which releases *Snrk2*s from the inhibition of *PP2C*s, and subsequently activates the downstream target ABRE-binding factor [34]. ABF could activate the promoter of the DRE-binding protein 2A, which has important functions in the osmotic stress response [35]. In this study, there was no significant difference in *ABF* expression between Tineke, Hiogi, and the control. However, in root tissue, upregulation of *PYR/PYL*, *Snrk2*, and *ABF* transcripts was induced by salt treatment. The number of *Snrk2* and *ABF* transcripts in Tineke was higher than in Hiogi, and this may have contributed to the greater salt tolerance of Tineke. Overexpression of *ABF* can enhance dehydration and stress tolerance via scavenging of ROS and modulating expression of stress-responsive genes [36]. In addition, HSPs play an important role in the ABF-induced ROS scavenging process [37], and the *HSP* expression observed in this study confirms this. GSH helps to alleviate salt stress in plants, so the damage from salt stress can be reduced with exogenous GSH supplementation [38]. The glutathione metabolism pathway was a critical pathway in Tineke after salt treatment. *GSH1* is a key gene in the production of glutathione synthetase, but no studies have reported that *GSH1* is related to salt tolerance in plants. However, we found 23 *GSH1* transcripts that were highly expressed in Tineke roots, and qPCR showed that the expression level of *GSH1* in the salt-tolerant varieties was significantly higher (*p<0*.*05*) than that of the salt-sensitive varieties. After salt treatment, the expression of *GSH1* significantly increased (*p<0*.*05*). Because *GSH1* is one of the key genes involved in the production of glutathione synthetase, high expression of *GSH1* could increase glutathione content, which promotes the glutathione cycle. Gamma-L-glutamyl-L-2-aminobutyrylglycine (ophthalmate, OPH) is a potential indicator of salt-tolerant rose varieties. However, due to the difficulties of OPH detection, the OPH content in the salt-tolerant Tineke variety was not determined in this study. In addition, plant glutathione transferases play an important role in the detoxification of xenobiotics and toxic lipid peroxides via regulation of the glutathione-binding reaction [39]. *GST* also has important functions in biochemical reactions of primary metabolism and secondary products such as flavonoid derivatives [40]. Plant cells are continuously stressed by toxic ROS generated by oxygen consumption during metabolic respiration. When plants are exposed to salt or drought stress, large amounts of ROS are generated [41]. The upregulation of *GST* can counterbalance the production of ROS, which would reduce the harm caused by abiotic stresses [42]. We found that the activity of GSTs in Tineke was sensitive to salt stress (47 transcripts were differentially expressed), but in Hiogi, only 14 differentially expressed transcripts were found. In addition, we found great differences in the expression of *GSH1* between the two *R*. *chinensis* cultivars when the plants were treated with salt, which has not been reported in previous salt stress studies. *GSH1* had been reported to be related to environmental stress [43, 44]. A stress-related post-transcriptional regulation was reported to related to the expression of *GSH1*, which further affects glutathione metabolism [45]. The overexpression of *GSH1* can promoted subsequent flowering and showed less responsive to SV treatment than in wild-type plant [46]. In this study, *GSH1* act as a response factor for salt stress. The high expression of *GSH1* has a certain linkage with salt tolerance. Where the *GSH1* can be used as a bio-marker of salt tolerance in Rosa chinensis, more work is needed to do. But in this study, we did discover the relationship between *GSH1* and salt tolerance. Chlorophyll content in plant leaf tissues typically decreases when subjected to salt stress [47]. In salt- and drought-resistant plants, the decline of chlorophyll content is not obvious, and in some species, chlorophyll levels may even increase [48]. The effects of salt stress on photosynthesis can be either direct or secondary, such as the oxidative stress arising from the superimposition of multiple stresses [49]. Chlorophyll levels in *R*. *chinensis* varieties suggested stronger salt tolerance in Tineke. An efficient response to the environment is particularly important for plants. In leaf tissue, when salt stimulation occurs, responses are triggered by primary osmotic stress signals or secondary signal metabolites (including hormones, ROS, and intracellular second messengers). These changes are consistent with our results. Salt and drought stress can induce stomatal closure and moderate increases in ROS levels, decreases in photosynthesis, and decreases in root growth rates [50]. The KEGG and GO enrichment results are again consistent with this. In Tineke, low concentrations of ROS maintained by plant hormone signal transduction and glutathione metabolism can reduce stomatal closure in leaves and maintain normal photosynthesis.

**Fig 9:**
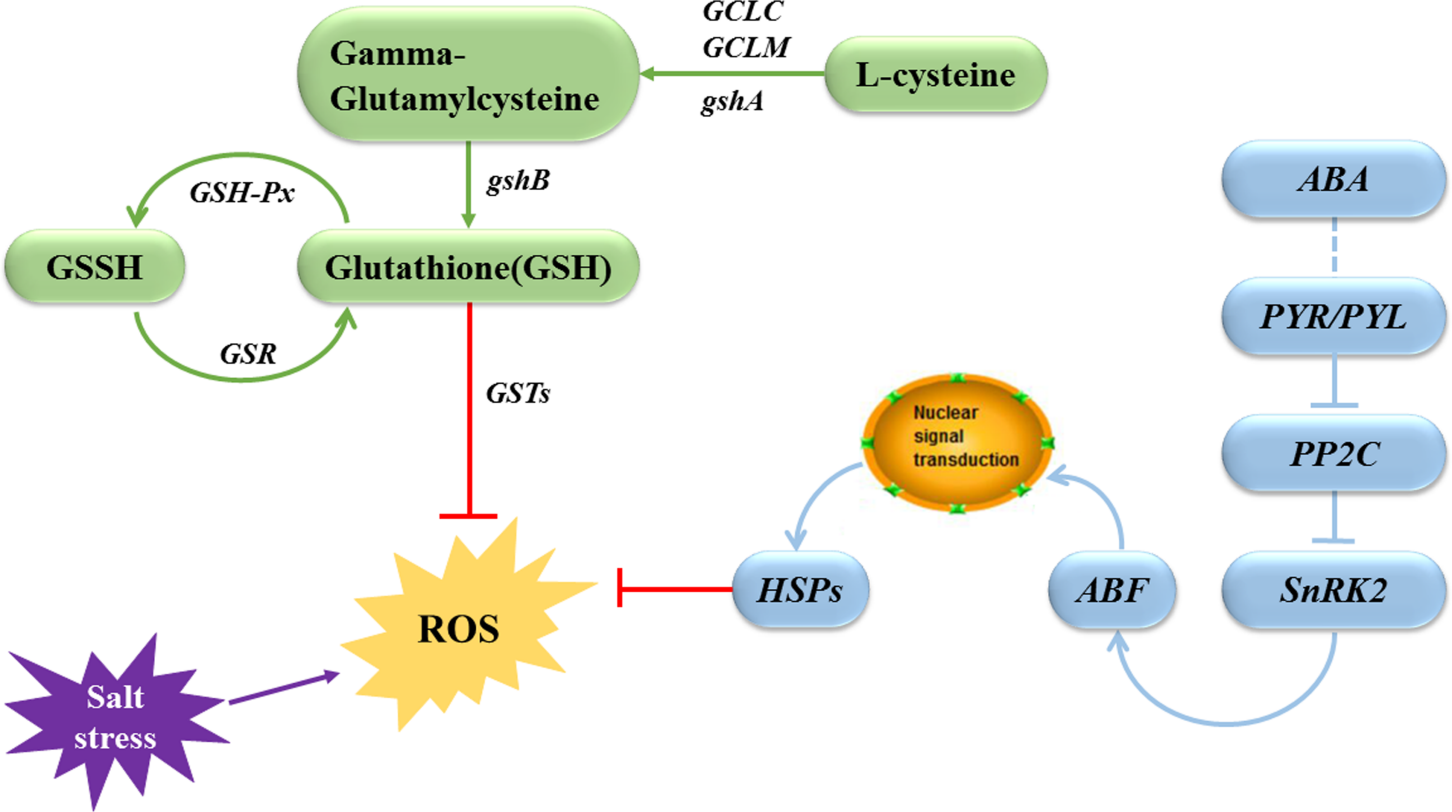
Schematic diagram of salt tolerance mechanism. *GSSH*: oxidized form glutathione; *GSR*: glutathione reductase; *GSH-Px*: Glutathione peroxidase; *GCLC*: Glutamate-Cysteine Ligase Catalytic Subunit; *GCLM*: glutamate-cysteine ligase modifier subunit.

In conclusion, the ABA-dependent signaling pathway was the main pathway involved in the salt stress responses of *R*. *chinensis*. Responses to salt tolerance were mainly modulated via two pathways: plant hormone signal transduction and glutathione metabolism (Fig 9). The difference in salt tolerance between the cultivars Hiogi and Tineke was due to differences in gene sensitivity to salt in these two pathways. The effects of salt stress in the roots are eventually manifested in the leaves, causing a series of changes in the leaves, including a reduced photosynthesis rate, which eventually causes leaf wilting. In Tineke, *Snrk2*, *ABF*, *HSP*, *GST*s, and *GSH1* showed strong activity under salt stress, and these genes are markers of salt tolerance.

## Supporting information

S1 Fig Venn diagram of DEGs in different comparison in leaf tissue. Different sets of colors represent different comparison.
(PDF)

S2 Fig Venn diagram of DEGs in different comparison in root tissue. Different sets of colors represent different comparison.
(PDF)

S1 Table Biological samples and grouping. (XLSX)

S2 Table Standard curves for SOD, PRO, and MDA. (XLSX)

S3 Table Standard curve for Na^+^ concentration in leaf tissue. (XLSX)

S4 Table CT value of *GAPDH*. (XLSX)

S5 Table Sequencing data summary. (XLSX)

S6 Table The top 20 GO enrichened terms. (XLSX)

S7 Table The top 20 KEGG enrichened terms. (XLSX)
