# Supplementary figures and images for: Genome-wide transcriptome analysis of the salt stress tolerance mechanism in Rosa chinensis
Source: PLoS One. 2018 Jul 26;13(7):e0200938. doi: 10.1371/journal.pone.0200938 (PMC6062038; doi:10.1371/journal.pone.0200938)

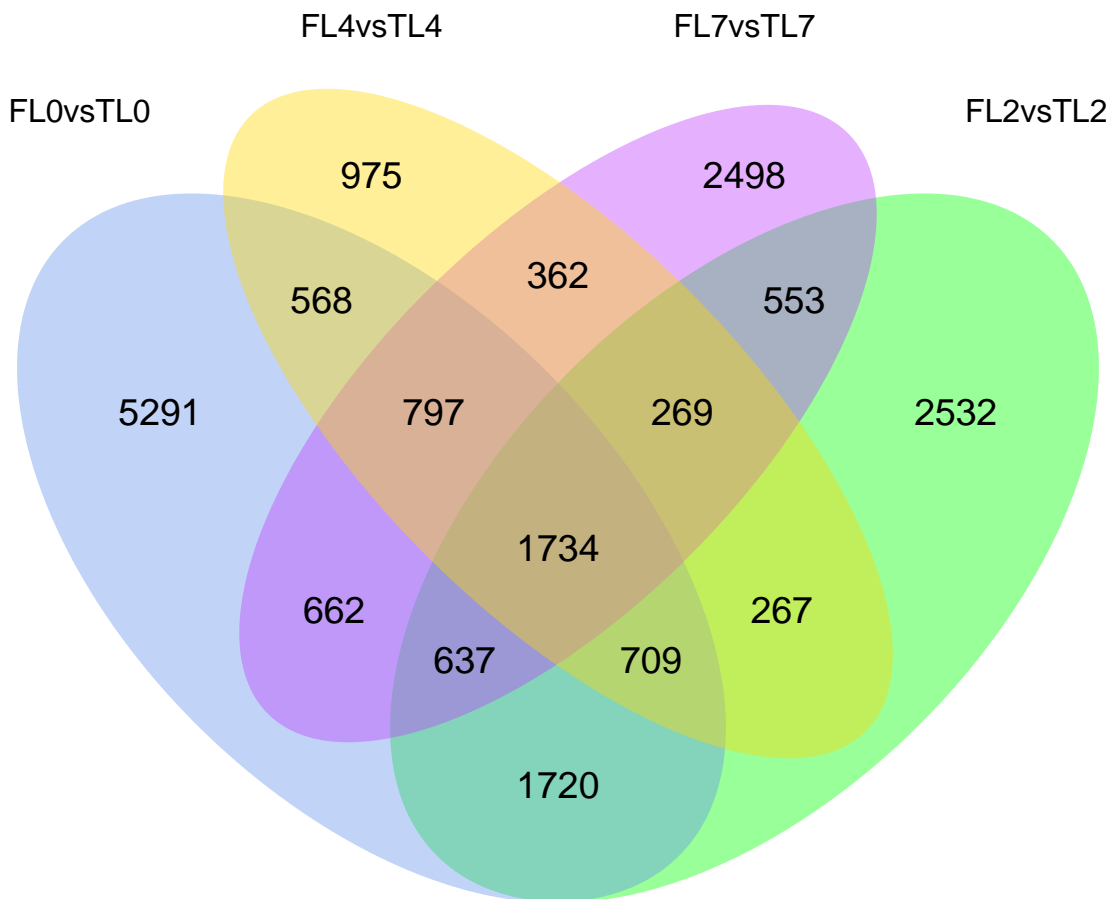

Supplement: S1 Fig — Different sets of colors represent different comparison. (PDF) [file pone.0200938.s001.pdf]

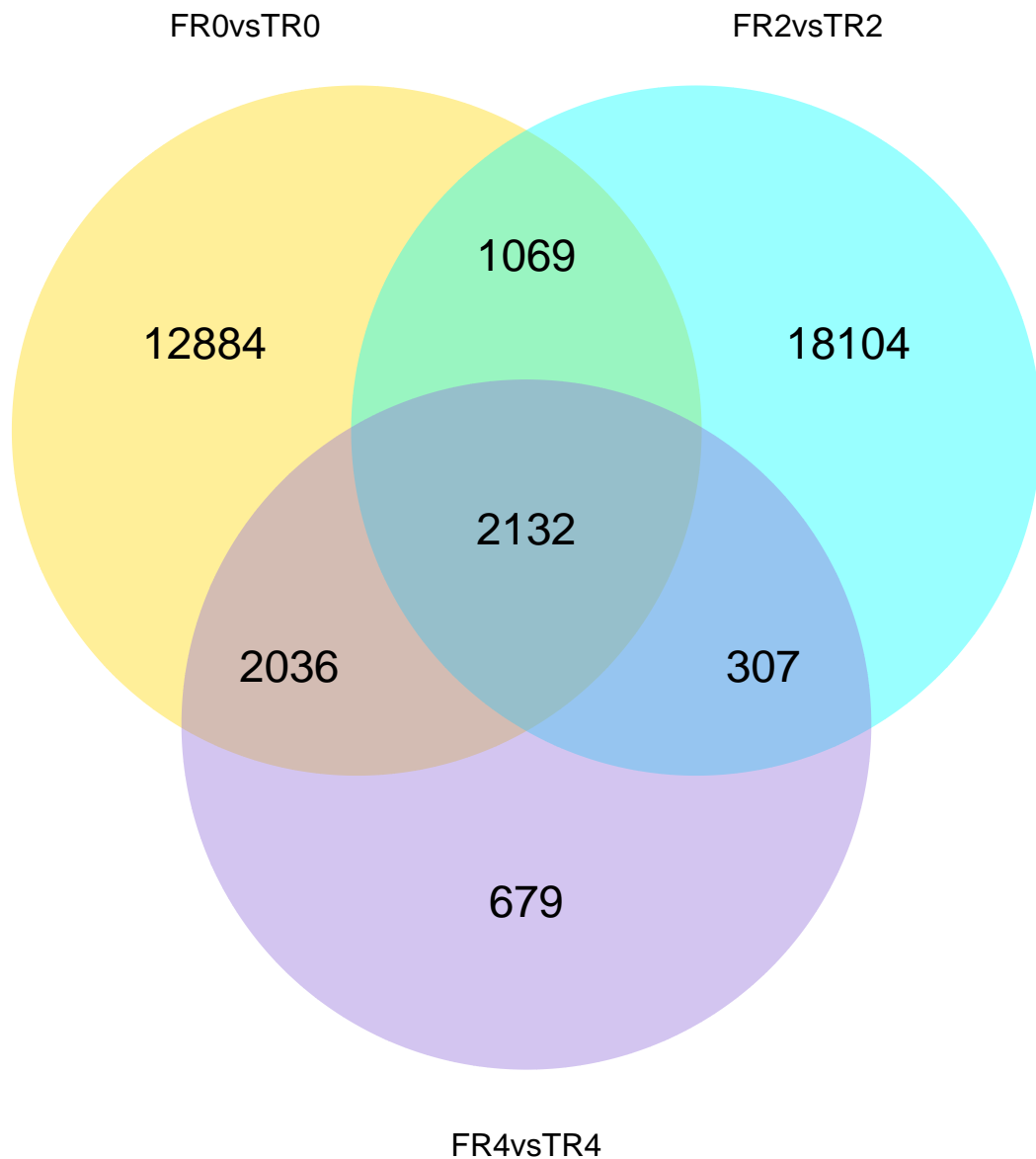

Supplement: S2 Fig — Different sets of colors represent different comparison. (PDF) [file pone.0200938.s002.pdf]
